# Increasing Potential Risk of a Global Aquatic Invader in Europe in Contrast to Other Continents under Future Climate Change

**DOI:** 10.1371/journal.pone.0018429

**Published:** 2011-03-30

**Authors:** Xuan Liu, Zhongwei Guo, Zunwei Ke, Supen Wang, Yiming Li

**Affiliations:** 1 Key Laboratory of Animal Ecology and Conservation Biology, Institute of Zoology, Chinese Academy of Sciences, Chaoyang, Beijing, China; 2 Graduate University of Chinese Academy of Sciences, Shijingshan, Beijing, China; National Institute of Water & Atmospheric Research, New Zealand

## Abstract

**Background:**

Anthropogenically-induced climate change can alter the current climatic habitat of non-native species and can have complex effects on potentially invasive species. Predictions of the potential distributions of invasive species under climate change will provide critical information for future conservation and management strategies. Aquatic ecosystems are particularly vulnerable to invasive species and climate change, but the effect of climate change on invasive species distributions has been rather neglected, especially for notorious global invaders.

**Methodology/Principal Findings:**

We used ecological niche models (ENMs) to assess the risks and opportunities that climate change presents for the red swamp crayfish (*Procambarus clarkii*), which is a worldwide aquatic invasive species. Linking the factors of climate, topography, habitat and human influence, we developed predictive models incorporating both native and non-native distribution data of the crayfish to identify present areas of potential distribution and project the effects of future climate change based on a consensus-forecast approach combining the CCCMA and HADCM3 climate models under two emission scenarios (A2a and B2a) by 2050. The minimum temperature from the coldest month, the human footprint and precipitation of the driest quarter contributed most to the species distribution models. Under both the A2a and B2a scenarios, *P. clarkii* shifted to higher latitudes in continents of both the northern and southern hemispheres. However, the effect of climate change varied considerately among continents with an expanding potential in Europe and contracting changes in others.

**Conclusions/Significance:**

Our findings are the first to predict the impact of climate change on the future distribution of a globally invasive aquatic species. We confirmed the complexities of the likely effects of climate change on the potential distribution of globally invasive species, and it is extremely important to develop wide-ranging and effective control measures according to predicted geographical shifts and changes.

## Introduction

The rapid spread of invasive species in combination with increasing globalization has threatened native biodiversity worldwide and resulted in great economic losses [1], [2]. Preventing the introduction of potential invaders is the most effective measure for the management of invasive species [3]. Another global environmental issue is the observation that climate change likely driven by human activities, most notably the increases in anthropogenic greenhouse gas emissions [4]. Climate change has complex effects on the potential distribution of non-native species by altering current climatic habitat [5], [6], [7]. It can create both greater invasion risks in regions where more areas become climatically suitable and/or reduced invasion risk in regions when areas currently at risk become climatically unsuitable. Some currently invaded lands may also become climatically unsuitable, potentially leading an invasive species to retreat from invaded areas [8]. Predictions of where climatically suitable habitats for invasive species are, or will be located in the future, and how the present habitats respond to climate change provide critical information for preventing biological invasions and the ecological restoration of invaded areas [9].

Aquatic ecosystems play an important role in the global biodiversity that provide a variety of valuable goods and services for human societies [10] and, compared to terrestrial ecosystems, are particularly subject to invasive species [11]. Aquatic invaders have been regarded as widespread dispersers because both hydrological network conduits and human activities can greatly facilitate their spread across large distances [12], [13]. In addition, aquatic ecosystems are very vulnerable to climate change, which can affect water runoff regimes, seasonality of water availability and air temperature, which in turn cycles directly back to changes in water temperature [14]. Furthermore, increasing the frequency of high-flow events due to climate change may result in more dispersal success and range expansion of aquatic invaders [15]. However, the impact of climate change on potential distributions of invasive aquatic species has been rather neglected [16].

Ecological niche models (ENMs) have been used widely to predict potential distributions of both native and non-native species [17], [18]. ENMs assume that the distribution of a species is in equilibrium with ecological conditions and that the niche of the species is conservative across space and time [19], [20]. For non-native species, ENMs have been used to capture species niches using current climate data from native distributions and have projected these niches onto potential invasive areas or future climate scenarios [21]. Recent studies have found that many non-native animals and plants can shift their climate niches from invaded ranges due to the release of historical and geographical constraints from native ranges or because of evolutionary changes in climate niche to new ranges [22], [23]. As a result, the ENMs based on native distributions only misrepresent the fundamental niche of non-native species and fail to correctly predict potential invasive distributions [24], [25]. A calibration of the ENMs based on comprehensive occurrence data from entire distributions, including both native ranges and invaded ranges, may provide a closer approximation of the fundamental niche and result in more thorough predictions of current and future distributions [23], [24], [25]. This process is particularly essential for those notorious invasive species that generally have wide and non-conservative niches [24]. It is also critical to predict distributions by incorporating both native and non-native data at the global scale for those worldwide notorious invaders under future climate change scenarios.

In the present study, we modeled the effects of climate change on potential global distributions of the invasive red swamp crayfish (*Procambarus clarkii*) using a maximum entropy model (MaxEnt), which was calibrated with occurrence data from both native and invaded ranges. This species is one of the most notorious globally invasive species in aquatic ecosystems [26] and has been recently recognized as one of the 100 “most invasive alien species in Europe” [27]. Niche shift has been shown in both this species and relevant invasive crayfish species [28], [29]. The red swamp crayfish is native to south-central United States and northeastern Mexico and has been introduced for human food and other uses on all continents, except Australia and Antarctica [30]. This species has the ability to travel long distances in most types of water courses and is able to move over land, which increases its range [31], [32], [33]. Both field and molecular evidence show that the expansion of *P. clarkii* can be accelerated by direct or indirect human-induced dispersions across natural barriers [34], [35]. The crayfish is an omnivorous consumer [36] mainly feeding on plants, insects, cladocerans, crayfish and organic sediment. Pre-adults and adults tend to be herbivorous, but juveniles seem to be predatory [37]. The crayfish can modify the existing food chains, leading to a rapid decrease in species richness (i.e., decreases in amphibian, waterfowl and native macroinvertebrate crayfish populations and destruction of plant coverage) and degradation of wetlands through the huge release of nutrients and direct consumption of macrophytes [38], [39], [40], [41]. Crayfish invasion can specifically result in native amphibian declines in water bodies by predation on eggs, tadpoles or adults [42], [43]. The crayfish can also have an ecological impact on water courses and crops, particularly rice crops. For example, some studies have shown that crayfish damage dams of agriculture and recreational water bodies due to its extensive burrowing activity [44]. Other studies have suggested that the crayfish can impact rice fields by interfering with the establishment of rice seedlings and the early stages of rice growth [45]. A number of studies have indicated that the crayfish is a vector of the crayfish fungus plague *Aphanomyces astaci* and an intermediate host for numerous parasites of vertebrates, including humans [46].

We first determined the relationship between factors including climate, altitude, vegetation index, human activity and habitat type, and the distribution of the crayfish and projected the potential distribution of the crayfish at a global scale using current climate data. We then projected global ranges of the crayfish under climate change based on a consensus-forecast approach of two atmosphere-ocean general circulation models (AOGCMs) with both A2a and B2a emission scenarios for potential future (2050) climates. Finally, we determined distributions of areas with expanded or reduced invasion risks or retreat areas based on present invaded land by overlapping the potential present and future distributions of the crayfish.

## Methods

### Occurrence Data

We collected the occurrence data of *P. clarkii* in Chinese waters based on extensive filed surveys during 2007 to 2010; we also included some related references (see Supporting Information Text S1). For other parts of the world, we used museum and literature records. The records from both the native and invasive ranges of *P. clarkii* were obtained from a variety of online databases including the GBIF, USGS, NMNH, NCSM and EUNIS, in addition to published literatures (see Supporting Information Text S1). Most of these records have precise geographical coordinates; however, a few records only have a general description of the locality. For these records, we assigned coordinates using published tools, such as Google Earth 5.0.1 (Google, Mountain View, USA), Global Gazetteer 2.2 (Falling Rain Genomics, Palo Alto, USA) and MapQuest (MapQuest Inc., Denver, USA). We excluded those locations that only provided provinces, counties and/or towns for which we could not obtain coordinate information using relevant mapping software. Our database contained a total of 1031 presence records imported into 2.5 arc-min grid cells around the world; 1002 records were retained for further analysis in MaxEnt after duplicates in same grid cells were removed. The processed coordinates were checked in the DIVA-GIS [47] by comparing the information provided with the records with locality (county, city) for biases and errors. We developed predictive models incorporating both native and non-native occurrence data on *P. clarkii* to identify present and future potential distributions. In addition, to reflect the response of invasive distributions to climate change, we excluded native ranges for the spatial analysis of range shifts under future conditions caused by climate change.

### Predictor Variables

We used eight eco-geographic factors, including climate, topography, habitats and human impacts that may potentially affect the *P. clarkii* distribution (Table 1). We used land-based climate variables obtained from the WorldClim database as a proxy for water climatic variables, as these models are generally considered to be correlated [48]. Compared with the marine habitats where there are more forcing parameters, this substitute of land-based climate data for aquatic climate data has been suggested to be acceptable in studies of freshwater systems such as lakes, streams, and rivers [49]. Land climates have been broadly used in most applications of ENMs across a wide taxa of freshwater species, including crayfish species [50], amphibians [51], turtles [52], fishes [53], snails [54] and diatom species [55]. Although a total of 19 global bioclimatic variables are available from WorldClim database [47], the precision of the predictions may be biased by the selection of predictive variables [25]. Inclusion of too many variables, especially for small sample sizes, could cause problems with over-fitting [18], [56], and physiological limits can influence the results, especially for invasive species [57]. Consequently, we first extracted all 19 bioclimatic data for 1000 random points from the geographic extent of the *P. clarkii* distributions modeled in DIVA-GIS. We then performed a Pearson correlation analysis on each climate variable for these points. For predictor pairs with a correlation coefficient of ≥|0.8|, we retained for modeling only those variables that influenced the known physiological constraints on the species' distributions based on previous studies [30], [33], [38], [40], [58], [59]. This resulted in a set of four largely independent climate variables including T*_max_*, T*_min_*, Prec*_dry_* and Prec*_wet_* from global climate point data (1950–2000) (Table 1). Our variable screening results also confirmed previous reports that these four climate variables had avoided the problem of multicollinearity issues [51]. Elevation data that were representative of the topography variable were also obtained from the WorldClim database. We included altitude as one predictor because it was identified as an important variable limiting *P. clarkii* distributions [30], [38]; in addition, altitude can act as a substitute for other environmental characteristics such as water velocity that are known to influence the species' distributions [60]. We obtained the land-cover data from the GLC 2000 database and reclassified the original land cover maps into two categories to facilitate interpretation: 1, grid cells with water and 0, without water. In addition, as it is suggested that hydrological microbial biomass that is estimated using a normalized difference vegetation index (NDVI) has a relationship with the *P. clarkii* distribution [61], we obtained and re-calculated the NDVI as the average of values for 12 months over an 18-year period from 1982 to 2000 using ArcGIS tools (ESRI, Redlands, CA, USA). Furthermore, we used the human footprint index, which is a composite factor of human influence on global surface that integrates data of land use, urbanization, population density, transportation networks and other human activities that are well known to facilitate species invasions, and is corrected by biome type [51], [52]. These data were based on nine geographical data sets that included vector maps, satellite images, and census data [62]. Human footprint, GLC and NDVI data were re-sampled to a resolution of 2.5 arc-min to match the bioclimatic variables using a bilinear interpolation function, which is considered to be more realistic than the simpler nearest-neighbor method [63].

**Table 1:** Description of factors included as predictor variables to model the effects of climate change on potential global distributions of the invasive *P. clarkii*.

Predictor Variable	Factor	Description
**Climate**	**T** ***_max_***	Average (1950–2000) max temperature (°C) of warmest month for 2.5 arc-min map grid cell; data available at http://www.worldclim.org
	**T** ***_min_***	Average (1950–2000) min temperature (°C) of coldest month for 2.5 arc-min map grid cell; data available at http://www.worldclim.org
	**Prec** ***_dry_***	Average (1950–2000) precipitation (mm) of driest quarter for 2.5 arc-min map grid cell; data available at http://www.worldclim.org
	**Prec** ***_wet_***	Average (1950–2000) precipitation (mm) of wettest quarter for 2.5 arc-min map grid cell; data available at http://www.worldclim.org
**Future Climate**	**T** ***_max_***_**,**_	Max temperature (°C) of the warmest month of the year 2050 provided by WorldClim for 2.5 arc-min map grid cell;
	**T** ***_min_***	Min temperature (°C) of the coldest month of the year 2050 by WorldClim for 2.5 arc-min map grid cell;
	**Prec** ***_dry_***	Precipitation (mm) of the driest quarter of the year 2050 provided by WorldClim for 2.5 arc-min map grid cell;
	**Prec** ***_wet_***	Precipitation (mm) of the wettest quarter of the year 2050 provided by WorldClim for 2.5 arc-min map grid cell;
**Topography**	**Altitude**	Height (m) above sea level; data available at http://www.worldclim.org
**Habitat**	**Land use**	Land cover type: water habitat (1) or not (0) reclassified from the original land cover maps; available at http://bioval.jrc.ec.europa.eu/products/glc2000/glc2000.php
	**NDVI**	Average (1982–2000) normalized difference vegetation index for 12 months; available at http://edit.csic.es/Soil-Vegetation-LandCover.html
**Human Impact**	**Human Footprint Index**	Human footprint index value (0–100) map grid cell; (data available at http://www.ciesin.columbia.edu/wild_areas/)

Future climate projections were based on two global general circulation models (AOGCMs), including the Canadian Climate Centre for Modeling and Analysis (CCCMA) and the Hadley Centre for Climate Prediction (HADCM3) under two Intergovernmental Panel on Climate Change Special Report Emissions Scenarios (A2a and B2a) for the year 2050 [4]. Future climate data were downloaded from the IPCC Third Assessment Report (TAR, available at www.worldclim.org/futdown.htm); these data are publicly available and have been calibrated and statistically downscaled in order to match the current conditions in the Worldclim database. We implemented an ArcInfo AML script (mkBCvars.aml, also available from the WorldClim database http://www.worldclim.org/mkBCvars.aml) to convert future high-resolution monthly climate data into the same bioclimatic variables that had been used for the current model construction (Table 1). These high-resolution bioclimatic variables derived from the TAR have been proven to be useful in scientific and policymaking communities. A2a and B2a scenarios are different in CO_2_ emissions, with A2a having medium to high emissions and B2a having low to medium emissions. Estimations of future human footprints, NDVI and land cover were not available. However, to decrease the risk of any misleading uncertainties by simple estimation [64], we assumed that these factors were constant as conservative respects for the future suitability. To reduce the uncertainty that can arise from different projections of circulation models, we used a consensus method for several models in the analysis and averaged the results [65], [66], [67]. The output predictions from two different AOGCMs, under both the A2a and B2a emission scenarios, were imported into ArcGIS software, and overlaid with equal weight using the raster calculator within the Spatial Analyst extension. The resulting data were used to generate the overall map indicating the distribution probabilities of crayfish on a global scale.

### Ecological Niche Modeling

We used MaxEnt (version 3.2; www.cs.princeton.edu/~schapire/maxent/) to model the environmental suitability of *P. clarkii* [63], [68]. MaxEnt is a machine learning method and is based on the estimates of species distributions obtained by finding the probability distribution using the maximum entropy principle [63]. In recent comparisons among several techniques of algorithms and predictions of species distributions, MaxEnt ranked as the best-performing model using presence-only data and showed a better performance comparable to 16 other algorithms, including several traditional tools using presence-absence data, such as general linear models (GLM) and general additive models (GAM) [69].

We developed models using the linear, quadratic, and hinge functions, which were considered as the best combination to avoid the problem of over-fitting [68], [70]. We generated models using 75% randomly assigned occurrences as a training dataset with the remaining 25% used as test dataset. We ran ten cross-validation replicates for each model and calculated the average AUC of the ten cross-validations (mean ± SD) to give a more robust estimate of predictive performance. We used the logistic output of MaxEnt with suitability values ranging from 0 (unsuitable habitat) to 1 (optimal habitat) [68]. We used the 10th percentile training presence as a suitability threshold, as suggested by Phillips and Dudik [68], i.e., we assumed that a grid cell was suitable if its suitability score was greater than the 10th percentile of training points. The choice of a suitability threshold can have a great effect on the probability map, and there is still no consensus on the use of “best” threshold to use [71], [72]. However, the 10th percentile threshold has been more commonly used (e.g. [51], [52], [73]) because it is considered to be highly conservative estimate of a species' tolerance to each predictor, considering the environmental complexity of the study area, and this threshold can therefore provide a more ecologically significant result [74], [75]. In addition, the 10th percentile threshold has been widely used because the true absence data has not been generally available [73], and other thresholds have usually been based on more complex methods requiring both presence and absence data [71], [72]. A jack-knife procedure was used to evaluate the relative importance of each predictor variable and the ability to correctly predict new ranges in the model [76]. Based on the test, the model was re-run by excluding each predictor, then a model was created using each variable in isolation. To reduce the impact of multiple presence within some grid cells due to more extensive sampling, we set MaxEnt to “remove duplicate presence records”.

### Model Robustness Validation

We used the following procedures to evaluate the predictive robustness of our modeling. Firstly, we used the area under the receiver operating characteristic curve (ROC) to measure the agreement between an observed species presence and its projected distribution [77]. The area under the curve (AUC) quantifies the model performance at all possible thresholds and is obtained by plotting positive cases against false-positive cases across a range of threshold values. AUC values can vary from 0.5, no better predictive ability than “random,” to 1.0, “perfect discrimination” [78]. However, the selection of background pseudo-absence points can have a significant impact on the result, and the high AUC value may result from the arbitrary selection of pseudo-absence data chosen from those regions outside the environmental tolerance of a species [79], [80]. To avoid this problem, we randomly selected the background points on a global scale that represented the water habitats that the *P. clarkii* could potentially inhabit using the GLC2000 database. Furthermore, we used null-models to test for significance of MaxEnt models and verified whether the models obtained differed significantly from what would be expected by chance, which is in accordance with the method of Raes and ter Steege [81]. For each model based on the crayfish distribution, we generated 99 null distributions of random localities without replacement from the geographical areas for which the *P. clarkii* distributions were modeled. The number of randomly drawn points per distribution was equal to the actual number of presence data. We then compared the AUC based on the random data with the AUC of the models generated using the actual *P. clarkii* distributions. Those randomly generated models were then used as a null-hypothesis for testing the significance of *P. clarkii* distribution models. The 95% confidence interval upper limit AUC value of this distribution was then compared with the AUC of the actual model to assess whether the *P. clarkii* models performed significantly better than expected by chance alone (using a significant level of 0.05). To increase the test accuracy of null-models, we followed the target-group method to draw ‘pseudo-presences', randomly selecting 99 times from the water grid cells on a global scale using GLC 2000 [80].

### Spatial Analysis of Climate Change Effects

There are generally two hypotheses to estimate the percentage loss or gain of species geographical ranges: the full dispersal hypothesis and the no dispersal hypothesis [64]. The full dispersal scenario assumes that a species can colonize all locations without physiological or environmental limitations, and the no dispersal hypothesis assumes that species will only persist in areas where the modeled historical and future geographical ranges overlap. In the present study, we assumed that *P. clarkii* would be able to move to the predicted habitats given its extensive dispersal capacity (for details, see Introduction).

We assessed the spatial patterns of distribution changes under climate change using the following approaches. We estimated the suitability for *P. clarkii* under both the current and future climate conditions for each pixel based on the 10th percentile training presence threshold of the MaxEnt output maps. We then quantified the potential range loss (currently suitable areas in terms of the number of 2.5 min grid cells projected to be lost), range gain (areas projected to become suitable), and range retained based on current potential ranges. To reflect the changes under future climate conditions, we estimated the species turnover, which was modified from β diversity metrics proposed by Cuesta-Camocho et al. [82] as follows: T = 100 × (L + G)/(CR + G), where T =  species turnover rate; G =  range gain; L =  range loss; and CR  =  current distribution. A turnover rate of 0 indicates that the species does not change between time slices, whereas a rate of 100 indicates that the future invasive patterns are completely different compared to the present conditions. Furthermore, to examine the spatial trends of distribution in response to climate change, we first extracted the latitude information of the center position of each grid cell that was suitable for *P. clarkii* distributions using spatial analysis tools in ArcGIS. We then categorized and summed the total range area (number of grid cells) per latitudinal band. Finally, we investigated the latitudinal pattern of the resulting gain or loss in range size on different continents under future climate change conditions.

## Results

### Model Performance

For all ten models, the ROC analyses revealed good performance by MaxEnt for both training AUC values (mean ± SD  = 0.9636±0.0002) and test AUC values (mean ± SD  = 0.9630±0.0017). The robustness of the model was also supported by null model tests, which showed that our model ranked first and scored significantly higher than the 99 null model AUCs (median  = 0.9191; 95% CI  = 0.9076 – 0.9305; P = 0.013).

### Eco-geographic Predictors and Current Predicted Distributions

**Figure 1:**
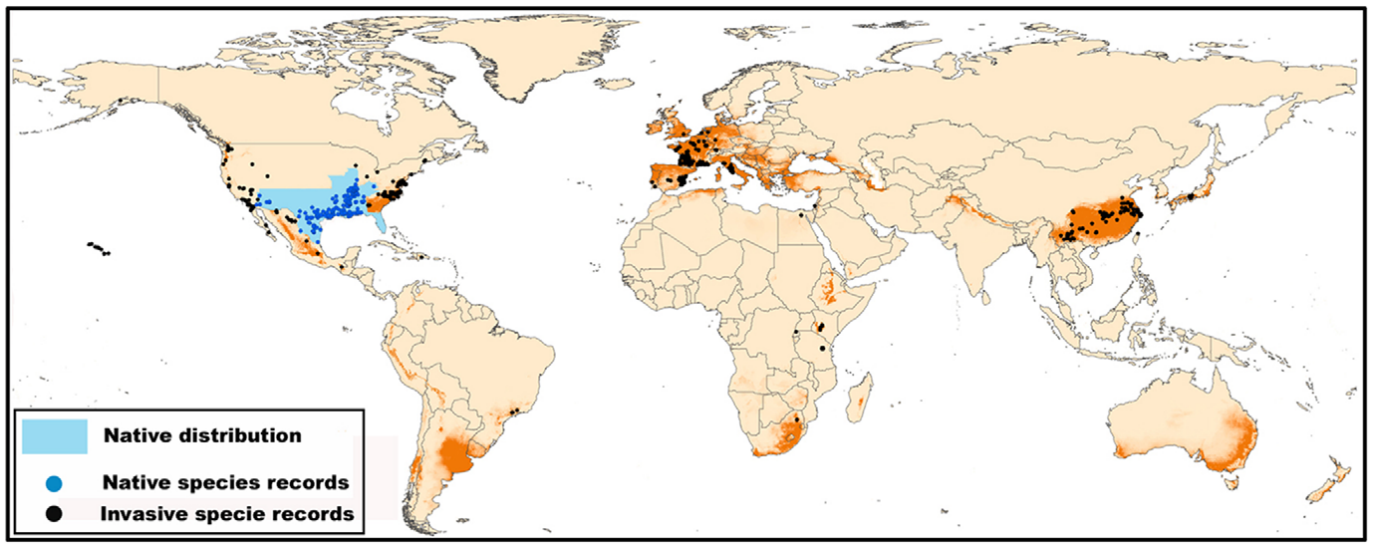
Worldwide projection for the present invasive potential of *P. clarkii*. The native distribution (blue shading) of *P. clarkii* and its occurrence is according to ISSG and published references (see Supporting Information Text S1). Black: non-native populations; blue: native populations.

The jack-knife procedure revealed that the distribution of *P. clarkii* is most constrained by T*_min_* (41.0% of explained variation accounted), followed by human footprint (26.7%), and Prec*_dry_* (18.7%) when used in isolation. The probability of occurrence was maximal for an intermediate T*_min_* ranging between -10°C and 9°C and a Prec*_dry_* ranging from 40 mm to 350 mm. Within America, the areas with the highest probability of *P. clarkii* distributions were mostly in the south-eastern United States, Mexico, Peru, central-eastern Argentina, western Chile and central Bolivia; for Asia, they were central-south China, Japan, a small proportion of northern Iran, northern areas across Pakistan, India and Nepal; for Africa, they were the eastern coastlines of South Africa, central Kenya, a small part of Algeria and Ethiopia; other areas were south-eastern Australia and the eastern coastline of New Zealand. Areas with high distribution probabilities ranged more widely in Europe, covering most areas of France, Belgium, Netherlands and Italy, central to northern Portugal, the eastern and northern part of Spain, southern United Kingdom, western Germany, central Serbia, northern Switzerland, southwestern and northern Greece and several eastern countries such as Turkey (Figure 1).

### Forecasted Range Shift Under Climate Change

**Figure 2:**
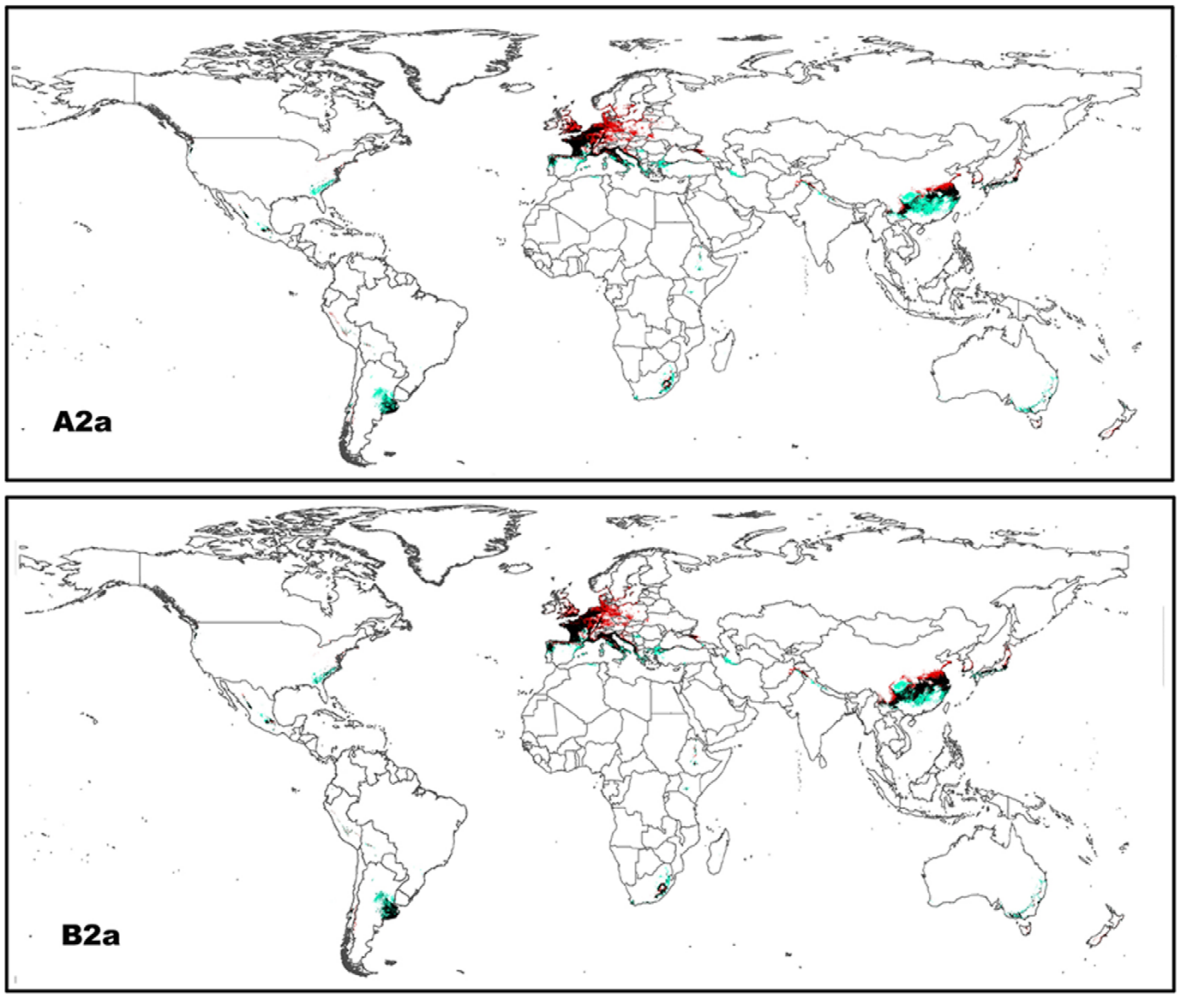
Projected impacts of climate change on future invasive potential of *P. clarkii.* Models were developed with a consensus-forecast approach using the CCCMA and HADCM3 climate models under two scenarios (A2a and B2a) by 2050: blue  =  current suitable areas projected to be lost with global climate change; black  =  current suitable areas projected to be retained; and red  =  areas projected to become suitable. The predicted suitability is based on the 10th percentile training presence threshold.

**Figure 3:**
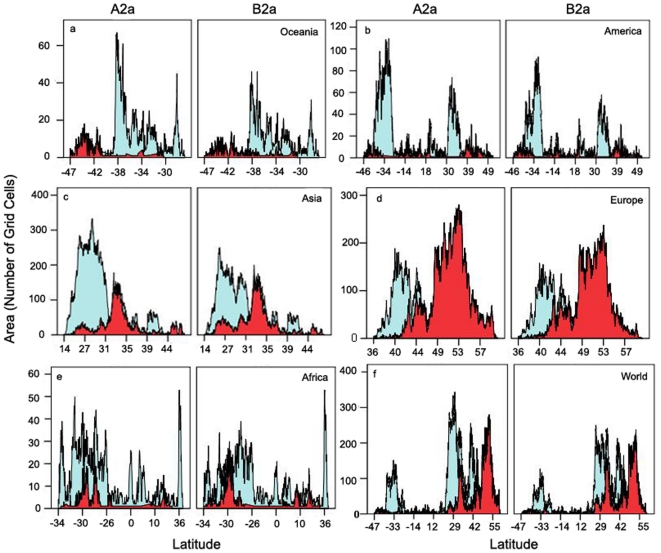
Climate change, biogeography and shift in range size of future invasive potential of *P. clarkii*. Projected latitudinal pattern of suitability changes between the present year and 2050 for two scenarios (A2a and B2a) with global climate change (blue, current suitable areas projected to be lost; and red, the proportion of areas projected to become suitable). The predicted suitability is based on the 10th percentile training presence threshold.

The future yielded projections with the A2a scenario were more extreme than the B2a scenario (Figure 2). Under the A2a scenario, there were less suitable habitats of *P. clarkii* retained or new habitats expanded, whereas more suitable habitats were lost when compared to the B2a scenario (Table 2). Ensemble global turnover rates were 58.3% and 45.6%, whereas 57.2% and 52.0% of invaded distributions led to a retreat under the A2a and B2a scenarios, respectively. The predicted future pattern was observed consistently under both scenarios: the effects of climate change on future suitability varied considerably among continents, with suitable areas increasing in Europe and the other four continents generally seeing a decrease under future climate conditions, with the greatest losses in potential area being for Africa (Table 2; Figure 3a–e). Several key regions with habitats that were suitable for new arrivals were mainly located in northern European countries, including Germany, Poland, Sweden and western Ukraine, a small part of eastern-north United States, some areas in eastern Afghanistan and in northern areas of China and Japan (Figure 2). In contrast, habitats would no longer exist in south-eastern United States, central areas of Mexico and Argentina for America, central areas of China, most areas of northern Iran, northern India and Nepal for Asia, eastern South Africa and northern Algeria for Africa, most of Greece, central Serbia and northwestern Turkey, large portions of eastern Portugal, southern areas of Italy, and small parts of central France for Europe, and most portions of southern-eastern Australia (Figure 2).

**Table 2:** Predicted changes (%) of potential suitable habitat of *P. clarkii* among five continents.

Habitat change	Europe	Africa	America	Asia	Oceania
**Habitat Retained**	49.0 (55.3)	30.6 (43.4)	38.9 (53.9)	37.0 (51.7)	40.3 (55.1)
**Habitat Gain**	38.3 (33.3)	10.1 (11.9)	8.3 (7.9)	16.5 (19.2)	10.4 (8.8)
**Habitat Loss**	12.7 (11.4)	59.3 (44.7)	52.8 (38.2)	46.5 (29.1)	49.3 (36.1)
**Turnover**	51.2 (44.9)	69.5 (55.6)	54.4 (41.7)	63.1 (48.0)	59.9 (45.1)

Models were developed with a consensus-forecast approach using the CCCMA and HADCM3 climate models under the A2a (B2a) scenarios by 2050. The predicted suitability is based on the 10th percentile training presence threshold.

The effects of climate change on global geographical shifts are discernible by shifting geographic distributions toward the poles (Figure 3f). Compared with the current distributions, future range gains mostly occurred in higher latitudes, whereas the reductions in future distributions were mainly concentrated in the mid-low latitudes, but Africa showed a somewhat mixed response (Figure 3a–e).

## Discussion

### Model Evaluation

For all ten cross-validation models, the fit results measured by the mean AUC value through the ROC analysis were high both for the training and test datasets. Using the approach of restricting ecologically meaningful background points, the AUC test is not likely to be biased by pseudo-absence ranges [79]. The use of null-model methodology and the significantly higher results than randomly generated models indicated that the relationship between species occurrence and selected predictors performed significantly better than expected at random [81]. Additionally, the use of a target-group procedure substantially improved the predictions of species distributions [80]. All of these results suggest a good fit of the models and the ability to discriminate from random chance was high [63]. We developed ENM predictive models by incorporating occurrence data from species' entire distributions and by providing a more thorough assessment of current and future ranges based on complex fundamental ecological niches [24], [25]. Compared with a recent prediction using relatively few presence records (n = 598) exploring the current potential distributions of *P. clarkii* on a global scale [60], our study found a similar pattern with highly suitable areas using a larger sample size (n = 1002), especially incorporating distribution information based on our four-year field surveys in China. In addition to the present potential distributions, we explored the future trends of *P. clarkii* invasions under climate change conditions, and our findings can be used for future conservation and management strategies of this global problematic invader.

The realized species distribution is the combined result of not only climate factors but also the consequences of other factors, such as anthropogenic effects, species interactions and stochastic events [83]. We built the current ENM covering predictors including climate, water habitats and human activity effects, which could lead to an approximate avoidance of omission and commission errors between real and predicted distributions [78]. Although these factors are not available and we kept them as a constant for future modeling, ENMs provide the first-order estimates of the climate variables that primarily constrain distributions [84] and potential distributions with climate change [85], [86]. To account for the fact that invasive species have the capacity to shift their climatic niches when introduced to novel regions and can occur in areas with different climatic conditions than those found in their native ranges [87], incorporating data from the *P. clarkii*'s entire distribution found in the present study will give a more complete assessment of current and future distributions [24]. Moreover, although factors, such as species interactions or stochastic events, are generally considered important for assessing invasion risk at local and landscape scales [88], the climate variable is expected to be more suitable for large-scale analyses of species range shifts, such as the present study [86]. In addition, the ROC analysis in MaxEnt based on presence and randomly selected background samples is recognized as one solution to such errors [63]. The high AUC value of the present study indicated that our models performed well and that their ability to differentiate suitable habitats from random chance was high. Nevertheless, the integration analysis at multiple spatial scales is considered as one important approach to understand how models at the global scale can be calibrated at the regional or local scales [19], [51]. It is therefore important to evaluate the role of small-scale factors influencing species distributions through a hierarchical framework in the future.

### Predictors and Current Distributions

*P. clarkii* is one of the most widely distributed invasive species in the world. Although previous studies have investigated its ecology at both local and regional scales, there is a lack of knowledge on its climatic requirements using data from the whole range. Our present study showed that there were close correlations between its presence and the two major climate variables of temperature and precipitation. The bell-shaped relationship observed between the crayfish distribution and both the minimum temperature of the coldest month and precipitation in dry quarters indicated that it would not be suitable for *P. clarkii* distribution in areas with very cold winters or very dry seasons, but some degree of seasonality is suitable over the whole range. Our findings have confirmed the plastic traits of this species that have been suggested by former studies [30], [33], [40]. A similar pattern was also observed in other widely distributed invasive species such as the American bullfrog [51]. These findings seem to imply a general rule that there is a greater environmental tolerance in those notorious invaders [89]. In the present study, we used land-based temperature and precipitation variables to model distributions of a freshwater invader on a global scale without using more complex water variables, such as the water chemistry data. We suggest that our modeled distributions should be tested in future studies at smaller spatial scales where more complex water parameters are available. These tests are especially important for marine species with more forcing parameters and complex factors that interact within land climatic variables [49]. The positive relationship between *P. clarkii* and human footprint not only supported the close relationship of this species with human activities [90] but also supplied global evidence that *P. clarkii* had the ability to colonize human-disturbed habitats that would be unsuitable for many native crayfish species [91]. Our results also appeared to support a general hypothesis that invasive species are more likely to use human-disturbed environments [92].

We identified several areas with maximum suitability for *P. clarkii* under the current climate, and these hotspots were highly in accordance with historical occurrence records (Figure 1). Although there were differences in climatically optimal regions among continents, our models indicated that all continents could support *P. clarkii* populations, and all have been invaded except Australia [30]. In contrast, we also observed that some areas predicted as being highly suitable were currently unoccupied. There are several possible hypotheses to explain this finding: for instance, there could be (1) some areas where the species is present but has not yet been detected; (2) invaded areas where the species has been successfully removed; (3) climatically suitable areas where the species has not yet spread; or (4) other factors such as species interactions not captured in the model that have limited its distributions.

### Potential Distributions Under Future Climate Change

Because projections of anthropogenically-induced global climate change will not end soon, the distribution response is likely to continue. Our study confirmed the complex effects of climate change on potential distributions of invasive species rather than simply promoting invasion risk [8], [9]. A total turnover rate exceeding 50% on average indicated that more than half of study areas would experience change in at least half of the current distribution. We found that some regions will become prone to invasion, some will reduce invasion risk by a decrease of invasive potentials, and some invaded areas will retreat as they will no longer be climatically suitable for *P. clarkii*. All of these findings will create both risks and opportunities for future *P. clarkii* invasions at the global scale. Under future climate conditions, some higher latitude areas are predicted to become more suitable for the invader, which is in accordance with previous studies on both plant and terrestrial animal species [5], [93]. There were contrasting projected changes in distributional areas of *P. clarkii* between Europe and other continents, where we predicted an increase and decreases, respectively. Given a concordant global pattern found in plant species [6], it may imply that Europe, especially northern European countries, will be more sensitive to invasive species and necessitate additional concern under future climate change. It has been suggested that the magnitude of future global warming is particularly higher in northern latitudes [94] such as northern Europe, and thus the likelihood of climate-induced range shifts of invasive species are more pronounced in such areas [16].

It is known that climate scenarios can induce different results from species distribution models [95], and the consensus-forecast approach is regarded as the most promising approach to overcome this problem [65], [66]. In the present study, two general global circulation models of CCCMA and HadCM3 and two different climate-change scenarios supplied by the TAR were used to give estimates of likely climatic changes in the coming decades. Our study using the consensus method greatly reduced the impact of variations between AOGCMs on prediction results. Future greenhouse gas emission scenarios can also influence the impacts of climate change [95]. We showed that scenario A2a generally made predictions of *P. clarkii* potential distributions with larger variations than the B2a scenario. This is not surprising, as the future potential distributions of *P. clarkii* will be more likely to be affected by higher greenhouse gas emissions.

As urbanization and human populations are still increasing, previous studies have suggested that human footprint (such as in Europe) is not likely to decrease in the near future [52]. Therefore, models assuming a constant human footprint in the present study are conservative with respect to the future suitability and invasion probability, which may increase even further. Additionally, another limitation of the present projected changes in the crayfish distribution is the barriers to crayfish dispersal. Although this species has both active and passive spread abilities, some natural and physical barriers could indeed hinder their dispersal at the regional scale [96]; the assumption that there would not be any constraint to their dispersal may be questionable in spite of the fact that it is a commonly made assumption in previous studies [53].

### Conservation and Management Implications

The complete eradication of invasive species once they have already become established and had significant impacts is extremely costly and often impossible, and therefore, prediction in early stages and subsequent preventions has widely been accepted as one of the most promising and cost-effective ways in managing invasive species [97]. In the present study, we have built a model to predict the invasion risk for the red swamp crayfish at the global scale, and our findings will be extremely important in developing wide-ranging monitoring and management strategies for future invasion risks.

We have found that both climate variables and anthropogenic disturbances can influence *P. clarkii* distributions. Our study has indicated that we should put forth future conservation and management measures combining these two aspects. For those regions with potentially suitable climates that have not been introduced yet, we suggest that the prevention of transport and release of species for human uses such as aquaculture, aquarium trade, recreation, biological control and teaching or research organisms should be seriously considered in future policies both through legislation and public education. For those areas with high invasion risk in which invasive species have been introduced for certain purposes, we suggest that it is of great importance to implement early detection and eradication plans and prohibit further introduction of the species in other sites. Previous studies have demonstrated the importance of improving aquaculture facilities to control and minimize the risk of species invasions [98]; we therefore suggest that appropriate cultivation facilities for stocking and aquaculture are important to prevent further *P. clarkii* invasions. For those areas already invaded, we suggest management strategies to highlight the role of landscape in controlling *P. clarkii* dispersal and focus on the construction of barriers by reducing their spread to more potentially suitable areas [96]. Possible retreated areas should also not be ignored because direct dispersal through water flow and indirect access to other suitable habitats by humans are still possible. In addition, areas that are no longer suitable for one invasive species may quickly become established by a new invasive species as the old is less competitive due to decreased suitability of the habitat [8]. These areas are ideal for exploring the response of native species to the disappearance of invasive species under climate change in the future.

## References

[1] Mack RN, Simberloff D, Lonsdale WM, Evans H, Clout M (2000). Biotic invasions: Causes, epidemiology, global consequences, and control.. Ecological Applications.

[2] Pimentel D, Zuniga R, Morrison D (2005). Update on the environmental and economic costs associated with alien-invasive species in the United States.. Ecological Economics.

[3] Simberloff D (2003). How much information on population biology is needed to manage introduced species?. Conservation Biology.

[4] IPCC (2001). http://ipcc-ddc.cru.uea.ac.uk.

[5] Roura-Pascual N, Suarez AV, Gomez C, Pons P, Touyama Y (2004). Geographical potential of Argentine ants (*Linepithema humile* Mayr) in the face of global climate change.. Proceedings of the Royal Society of London Series B-Biological Sciences.

[6] Peterson AT, Stewart A, Mohamed KI, Araújo MB (2008). Shifting Global Invasive Potential of European Plants with Climate Change.. PLoS ONE.

[7] Thuiller W, Richardson DM, Pysek P, Midgley GF, Hughes GO (2005). Niche-based modelling as a tool for predicting the risk of alien plant invasions at a global scale.. Global Change Biology.

[8] Bradley BA, Oppenheimer M, Wilcove DS (2009). Climate change and plant invasions: restoration opportunities ahead?. Global Change Biology.

[9] Walther GR, Roques A, Hulme PE, Sykes MT, Pysek P (2009). Alien species in a warmer world: risks and opportunities.. Trends in Ecology & Evolution.

[10] Dudgeon D, Arthington AH, Gessner MO, Kawabata ZI, Knowler DJ (2006). Freshwater biodiversity: importance, threats, status and conservation challenges.. Biological Reviews.

[11] Cox JG, Lima SL (2006). Naivete and an aquatic-terrestrial dichotomy in the effects of introduced predators.. Trends in Ecology & Evolution.

[12] Bohonak AJ, Jenkins DG (2003). Ecological and evolutionary significance of dispersal by freshwater invertebrates.. Ecology Letters.

[13] Rahel FJ (2007). Biogeographic barriers, connectivity and homogenization of freshwater faunas: it's a small world after all.. Freshwater Biology.

[14] Woodward G, Perkins DM, Brown LE (2010). Climate change and freshwater ecosystems: impacts across multiple levels of organization.. Philosophical Transactions of the Royal Society B-Biological Sciences.

[15] Truscott AM, Soulsby C, Palmer SCF, Newell L, Hulme PE (2006). The dispersal characteristics of the invasive plant Mimulus guttatus and the ecological significance of increased occurrence of high-flow events.. Journal of Ecology.

[16] Rahel FJ, Olden JD (2008). Assessing the effects of climate change on aquatic invasive species.. Conservation Biology.

[17] Araújo MB, Guisan A (2006). Five (or so) challenges for species distribution modelling.. Journal of Biogeography.

[18] Heikkinen RK, Luoto M, Araújo MB, Virkkala R, Thuiller W (2006). Methods and uncertainties in bioclimatic envelope modelling under climate change.. Progress in Physical Geography.

[19] Guisan A, Thuiller W (2005). Predicting species distribution: offering more than simple habitat models.. Ecology Letters.

[20] Peterson A, Holt R (2003). Niche differentiation in Mexican birds: using point occurrences to detect ecological innovation.. Ecology Letters.

[21] Mau-Crimmins TM, Schussman HR, Geiger EL (2006). Can the invaded range of a species be predicted sufficiently using only native-range data? Lehmann lovegrass (*Eragrostis lehmanniana*) in the southwestern United States.. Ecological Modelling.

[22] Broennimann O, Treier UA, Muller-Scharer H, Thuiller W, Peterson AT (2007). Evidence of climatic niche shift during biological invasion.. Ecology Letters.

[23] Broennimann O, Guisan A (2008). Predicting current and future biological invasions: both native and invaded ranges matter.. Biology Letters.

[24] Beaumont LJ, Gallagher RV, Thuiller W, Downey PO, Leishman MR (2009). Different climatic envelopes among invasive populations may lead to underestimations of current and future biological invasions.. Diversity and Distributions.

[25] Rödder D, Lötters S (2009). Niche shift versus niche conservatism? Climatic characteristics of the native and invasive ranges of the Mediterranean house gecko (*Hemidactylus turcicus*).. Global Ecology and Biogeography.

[26] Ilhéu M, Bernardo J, Fernandes S (2007). Predation of invasive crayfish on aquatic vertebrates: the effect of *Procambarus clarkii* on fish assemblages in Mediterranean temporary streams..

[27] Drake JA (2009). Handbook of alien species in Europe..

[28] Correia AM (2002). Niche breadth and trophic diversity: feeding behaviour of the red swamp crayfish (*Procambarus clarkii*) towards environmental availability of aquatic macroinvertebrates in a rice field (Portugal).. Acta Oecologica.

[29] Olsson K, Stenroth P, Nystrom P, Graneli W (2009). Invasions and niche width: does niche width of an introduced crayfish differ from a native crayfish?. Freshwater Biology.

[30] Huner J, Holdich DM, Lowery RS (1988). *Procambarus* in North America and elsewhere.. Freshwater crayfish: Biology, management and exploitation.

[31] Aquiloni L, Ilheu M, Gherardi F (2005). Habitat use and dispersal of the invasive crayfish *Procambarus clarkii* in ephemeral water bodies of Portugal.. Marine and Freshwater Behaviour and Physiology.

[32] Gherardi F, Barbaresi S, Salvi G (2000). Spatial and temporal patterns in the movement of *Procambarus clarkii*, an invasive crayfish.. Aquatic Sciences.

[33] Cruz MJ, Rebelo R (2007). Colonization of freshwater habitats by an introduced crayfish, *Procambarus clarkii*, in Southwest Iberian Peninsula.. Hydrobiologia.

[34] Barbaresi S, Gherardi F (2000). The invasion of the alien crayfish *Procambarus clarkii* in Europe, with particular reference to Italy.. Biological Invasions.

[35] Yue GH, Li JL, Bai ZY, Wang CM, Feng FLC (2010). Genetic diversity and population structure of the invasive alien red swamp crayfish.. Biological Invasions.

[36] Smart AC, Harper DM, Malaisse F, Schmitz S, Coley S (2002). Feeding of the exotic Louisiana red swamp crayfish, *Procambarus clarkii* (Crustacea, Decapoda), in an African tropical lake: Lake Naivasha, Kenya.. Hydrobiologia.

[37] Correia AM (2003). Food choice by the introduced crayfish *Procambarus clarkii*.. Annales Zoologici Fennici.

[38] Gil-Sanchez JM, Alba-Tercedor J (2002). Ecology of the native and introduced crayfishes Austropotamobius pallipes and *Procambarus clarkii* in southern Spain and implications for conservation of the native species.. Biological Conservation.

[39] Rodriguez CF, Becares E, Fernandez-Alaez M, Fernandez-Alaez C (2005). Loss of diversity and degradation of wetlands as a result of introducing exotic crayfish.. Biological Invasions.

[40] Geiger W, Alcorlo P, Baltanas A, Montes C (2005). Impact of an introduced Crustacean on the trophic webs of Mediterranean wetlands.. Biological Invasions.

[41] Gherardi F, Acquistapace P (2007). Invasive crayfish in Europe: the impact of *Procambarus clarkii* on the littoral community of a Mediterranean lake.. Freshwater Biology.

[42] Witte CL, Sredl MJ, Kane AS, Hungerford LL (2008). Epidemiologic analysis of factors associated with local disappearances of native ranid frogs in Arizona.. Conservation Biology.

[43] Cruz M, Rebelo R (2005). Vulnerability of Southwest Iberian amphibians to an introduced crayfish, *Procambarus clarkii*.. Amphibia-Reptilia.

[44] Correia AM, Ferreira O (1995). Burrowing Behavior of the Introduced Red Swamp Crayfish *Procambarus Clarkii* (Decapoda, Cambaridae) in Portugal.. Journal of Crustacean Biology.

[45] Anastácio P, Marques J (1997). Crayfish, *Procambarus clarkii*, effects on initial stages of rice growth in the lower Mondego River valley (Portugal).. Freshwater crayfish.

[46] Gherardi F (2007). Understanding the impact of invasive crayfish..

[47] Hijmans RJ, Cameron SE, Parra JL, Jones PG, Jarvis A (2005). Very high resolution interpolated climate surfaces for global land areas.. International Journal of Climatology.

[48] Caissie D (2006). The thermal regime of rivers: a review.. Freshwater Biology.

[49] Lasram FB, Guilhaumon F, Albouy C, Somot S, Thuiller W (2010). The Mediterranean Sea as a ‘cul-de-sac’ for endemic fishes facing climate change.. Global Change Biology.

[50] Capinha C, Anastácio P (2010). Assessing the environmental requirements of invaders using ensembles of distribution models.. Diversity and Distributions.

[51] Ficetola GF, Thuiller W, Miaud C (2007). Prediction and validation of the potential global distribution of a problematic alien invasive species - the American bullfrog.. Diversity and Distributions.

[52] Ficetola GF, Thuiller W, Padoa-Schioppa E (2009). From introduction to the establishment of alien species: bioclimatic differences between presence and reproduction localities in the slider turtle.. Diversity and Distributions.

[53] Buisson L, Thuiller W, Lek S, Lim P, Grenouillet G (2008). Climate change hastens the turnover of stream fish assemblages.. Global Change Biology.

[54] Cordellier M, Pfenninger M (2008). Climate-driven range dynamics of the freshwater limpet, *Ancylus fluviatilis* (Pulmonata, Basommatophora).. Journal of Biogeography.

[55] Kumar S, Spaulding SA, Stohlgren TJ, Hermann KA, Schmidt TS (2009). Potential habitat distribution for the freshwater diatom *Didymosphenia geminata* in the continental US.. Frontiers in Ecology and the Environment.

[56] Beaumont LJ, Hughes L, Poulsen M (2005). Predicting species distributions: use of climatic parameters in BIOCLIM and its impact on predictions of species' current and future distributions.. Ecological Modelling.

[57] Aragón P, Baselga A, Lobo JM (2010). Global estimation of invasion risk zones for the western corn rootworm *Diabrotica virgifera virgifera*: integrating distribution models and physiological thresholds to assess climatic favourability.. Journal of Applied Ecology.

[58] Diéguez-Uribeondo J, Rueda A, Castien E, Bascones J (1997). A plan of restoration in Navarra for the native freshwater crayfish species of Spain, *Austropotamobius pallipes*.. Bulletin Francais De La Peche Et De La Pisciculture.

[59] Wang SP, Ke ZW, Cai YF, Li YM (2010). Distribution of introduced red swamp crayfish (*Procambarus clarkii*) in permanent lentic water bodies on Zhoushan Archipelago of China and related affecting factors.. Chinese Journal of Ecology.

[60] Capinha C, Leung B, Anastácio P (2010). Predicting worldwide invasiveness for four major problematic decapods: an evaluation of using different calibration sets.. http://dx.doi.org/10.1111/j.1600-0587.2010.06369.x.

[61] Poulin B, Lefebvre G, Crivelli AJ (2007). The invasive red swamp crayfish as a predictor of Eurasian bittern density in the Camargue, France.. Journal of Zoology.

[62] Sanderson EW, Jaiteh M, Levy MA, Redford KH, Wannebo AV (2002). The human footprint and the last of the wild.. Bioscience.

[63] Phillips SJ, Anderson RP, Schapire RE (2006). Maximum entropy modeling of species geographic distributions.. Ecological Modelling.

[64] Thuiller W, Broennimann O, Hughes G, Alkemade JRM, Midgley GF (2006). Vulnerability of African mammals to anthropogenic climate change under conservative land transformation assumptions.. Global Change Biology.

[65] Thuiller W (2004). Patterns and uncertainties of species' range shifts under climate change.. Global Change Biology.

[66] Marmion M, Parviainen M, Luoto M, Heikkinen RK, Thuiller W (2009). Evaluation of consensus methods in predictive species distribution modelling.. Diversity and Distributions.

[67] Araújo MB, Pearson RG (2005). Equilibrium of species' distributions with climate.. Ecography.

[68] Phillips SJ, Dudik M (2008). Modeling of species distributions with Maxent: new extensions and a comprehensive evaluation.. Ecography.

[69] Elith J, Graham CH, Anderson RP, Dudik M, Ferrier S (2006). Novel methods improve prediction of species' distributions from occurrence data.. Ecography.

[70] Peterson AT, Papes M, Eaton M (2007). Transferability and model evaluation in ecological niche modeling: a comparison of GARP and Maxent.. Ecography.

[71] Liu CR, Berry PM, Dawson TP, Pearson RG (2005). Selecting thresholds of occurrence in the prediction of species distributions.. Ecography.

[72] Jimenez-Valverde A, Lobo JM (2007). Threshold criteria for conversion of probability of species presence to either-or presence-absence.. Acta Oecologica-International Journal of Ecology.

[73] Brito JC, Santos X, Pleguezuelos JM, Sillero N (2008). Inferring evolutionary scenarios with geostatistics and geographical information systems for the viperid snakes *Vipera latastei* and *Vipera monticola*.. Biological Journal of the Linnean Society.

[74] Svenning JC, Normand S, Kageyama M (2008). Glacial refugia of temperate trees in Europe: insights from species distribution modelling.. Journal of Ecology.

[75] Jarnevich CS, Reynolds LV (2011). Challenges of predicting the potential distribution of a slow-spreading invader: a habitat suitability map for an invasive riparian tree.. Biological Invasions.

[76] Pearson RG, Raxworthy CJ, Nakamura M, Peterson AT (2007). Predicting species distributions from small numbers of occurrence records: a test case using cryptic geckos in Madagascar.. Journal of Biogeography.

[77] Manel S, Williams HC, Ormerod SJ (2001). Evaluating presence-absence models in ecology: the need to account for prevalence.. Journal of Applied Ecology.

[78] Fielding AH, Bell JF (1997). A review of methods for the assessment of prediction errors in conservation presence/absence models.. Environmental Conservation.

[79] VanDerWal J, Shoo LP, Graham C, William SE (2009). Selecting pseudo-absence data for presence-only distribution modeling: How far should you stray from what you know?. Ecological Modelling.

[80] Phillips SJ, Dudik M, Elith J, Graham CH, Lehmann A (2009). Sample selection bias and presence-only distribution models: implications for background and pseudo-absence data.. Ecological Applications.

[81] Raes N, ter Steege H (2007). A null-model for significance testing of presence-only species distribution models.. Ecography.

[82] Cuesta-Camocho F, Ganzenmuller A, Peralvo M, Novoa J, Riofrio G (2006). Predicting species's niche distribution shifts and biodiversity change within climate change scenarios: a regional assessment for bird and plant species in the northern tropical Andes..

[83] Dormann CF (2007). Promising the future? Global change projections of species distributions.. Basic and Applied Ecology.

[84] Hirzel AH, Hausser J, Chessel D, Perrin N (2002). Ecological-niche factor analysis: How to compute habitat-suitability maps without absence data?. Ecology.

[85] Araújo MB, Pearson RG, Thuiller W, Erhard M (2005). Validation of species-climate impact models under climate change.. Global Change Biology.

[86] Hijmans RJ, Graham CH (2006). The ability of climate envelope models to predict the effect of climate change on species distributions.. Global Change Biology.

[87] Gallagher RV, Beaumont LJ, Hughes L, Leishman MR (2010). Evidence for climatic niche and biome shifts between native and novel ranges in plant species introduced to Australia.. Journal of Ecology.

[88] Bradley BA, Mustard JF (2006). Characterizing the landscape dynamics of an invasive plant and risk of invasion using remote sensing.. Ecological Applications.

[89] Sakai AK, Allendorf FW, Holt JS, Lodge DM, Molofsky J (2001). The population biology of invasive species.. Annual Review of Ecology and Systematics.

[90] Barbaresi S, Santini G, Tricarico E, Gherardi F (2004). Ranging behaviour of the invasive crayfish, *Procambarus clarkii* (Girard).. Journal of Natural History.

[91] Paglianti A, Gherardi F (2004). Combined effects of temperature and diet on growth and survival of young-of-year crayfish: A comparison between indigenous and invasive species.. Journal of Crustacean Biology.

[92] Nilsson C, Berggren K (2000). Alterations of riparian ecosystems caused by river regulation.. Bioscience.

[93] Walther GR, Gritti ES, Berger S, Hickler T, Tang ZY (2007). Palms tracking climate change.. Global Ecology and Biogeography.

[94] ACI (2005). Arctic climate impact assessment..

[95] Mckenney DW, Pedlar JH, Lawrence K, Campbell K, Hutchinson MF (2007). Potential impacts of climate change on the distribution of North American trees.. Bioscience.

[96] Kerby JL, Riley SPD, Kats LB, Wilson P (2005). Barriers and flow as limiting factors in the spread of an invasive crayfish (*Procambarus clarkii*) in southern California streams.. Biological Conservation.

[97] Hulme PE (2006). Beyond control: wider implications for the management of biological invasions.. Journal of Applied Ecology.

[98] Liu X, Li Y (2009). Aquaculture enclosures relate to the establishment of feral populations of introduced species.. PLoS ONE.

